# Influence of Host Blood Meal Source on Gut Microbiota of Wild Caught *Aedes aegypti*, a Dominant Arboviral Disease Vector

**DOI:** 10.3390/microorganisms10020332

**Published:** 2022-02-01

**Authors:** Devojit Kumar Sarma, Manoj Kumar, Jigyasa Dhurve, Namrata Pal, Poonam Sharma, Meenu Mariya James, Deepanker Das, Sweta Mishra, Swasti Shubham, Manoj Kumawat, Vinod Verma, Rajnarayan R. Tiwari, Ravinder Nagpal, Francesco Marotta

**Affiliations:** 1 ICMR—National Institute for Research in Environmental Health, Bhopal Bypass Road, Bhouri, Bhopal 462030, Madhya Pradesh, India; manoj15ndri@gmail.com (M.K.); jigyasa.dhurve47@gmail.com (J.D.); namratapal017@gmail.com (N.P.); poonam.mannan91@gmail.com (P.S.); meenumariya07@gmail.com (M.M.J.); deepankernireh@gmail.com (D.D.); msweta6@yahoo.in (S.M.); swasti.shubham@gmail.com (S.S.); manojkbiochem@gmail.com (M.K.); rajtiwari2810@yahoo.co.in (R.R.T.); 2 Stem Cell Research Centre, Department of Hematology, Sanjay Gandhi Post-Graduate Institute of Medical Sciences, Lucknow 226014, Uttar Pradesh, India; vverma29@gmail.com; 3 Department of Nutrition & Integrative Physiology, College of Health & Human Sciences, Florida State University, Tallahassee, FL 32306, USA; rnagpal@fsu.edu; 4 ReGenera R&D International for Aging Intervention, 20144 Milano, Lombardia, Italy

**Keywords:** 16S metagenomics, *Aedes aegypti*, blood-feeding behavior, gut microbiota, vector-borne diseases

## Abstract

Blood feeding is an important behavior of *Aedes aegypti*, a dominant arboviral disease vector, as it can establish and transmit viruses to humans. Bacteria associated with the mosquito gut can modulate the biological characteristics and behavior of disease vectors. In this study, we characterized the gut microbiota composition of human-blood-fed (HF), non-human-blood-fed (NHF) and non-fed (NF) field-collected *Ae. aegypti* mosquitoes, using a 16S metagenomic approach, to assess any association of bacterial taxa with the blood-feeding behavior of *Ae. aegypti.* A significant difference in the microbiota composition between the HF and NF mosquito group was observed. A significant association was observed in the relative abundance of families *Rhodobacteraceae, Neisseriaceae* and *Dermacoccaceae* in the HF group in contrast to NF and NHF *Ae. aegypti* mosquitoes, respectively. At the class level, two classes (*Rhodobacterales* and *Neisseriales*) were found to be in higher abundance in the HF mosquitoes compared to a single class of bacteria (*Caulobacterales*) in the NF mosquitoes. These results show that human-blood feeding may change the gut microbiota in wild *Ae. aegypti* populations. More research is needed to determine how changes in the midgut bacterial communities in response to human-blood-feeding affect the vectorial capacity of *Ae. aegypti.*

## 1. Introduction

Mosquitoes are unquestionably the most studied groups of insects with public health importance due to their involvement in the transmission of several diseases. Globally, 80% of the population is at the risk of one or more vector-borne diseases (VBD) [1], accounting for more than 17% of all infectious diseases and causing approximately 1 million deaths annually. The maintenance and transmission of the pathogens causing malaria, lymphatic filariasis, and numerous viral infections such as dengue virus (DENV), chikungunya virus (CHIKV) and the recent Zika virus (ZIKV) outbreak is absolutely dependent on the availability of competent mosquito vectors and their intrinsic capability to feed on human blood [2]. Mosquitoes’ blood-feeding habit not only provides them with critical blood proteins and nutrients for egg production and reproductive fitness, but it also allows pathogens to establish themselves in and be transmitted to humans by their arthropod hosts [2,3]. Therefore, both blood-feeding behavior and blood meal host preference are critical as an increased biting rate leads to an increased vectorial capacity wherein the vector has more chances to acquire and spread pathogens [4]. However, studies have revealed that the blood quality, and thus host species, can alter reproductive output, suggesting that disease vectors have a specialized blood-feeding behavior [5]. The fact that most disease-causing agents transmitted by mosquitoes are host-specific (*Plasmodium falciparum*, *Plasmodium vivax*, *Wuchereria bancrofti*, DENV) indicates that their survival is largely dependent on their ability to transmit from one individual to another, which is aided by mosquito vectors. It was also observed that specific host species contribute disproportionately to pathogen transmission due to differences in number, exposure, and susceptibility to infection [6]. The intensity and peak timing of West Nile Virus (WNV) infection rates in the Northern house mosquito *Culex pipens pipens*, which was predominantly driven by its exclusive preference for feeding on *Turdus migratorius* (American robin), demonstrated the same [7]. Therefore, host selection patterns of blood-feeding arthropods are important determinants of epidemiological dynamics for vector-borne diseases, and they are generally applied to understand the natural transmission dynamics, predict disease risk, and inform disease surveillance and control efforts. The impact of vector host-feeding choices on arthropod gut microbial communities is a critical yet unexplored aspect that has the ability to change vector-borne disease dynamics. Some bacterial species present in the mosquito gut were reported to influence a variety of biological and physiological features, including mosquito longevity and insecticide susceptibility [8,9], reproductive fitness [10] and can modulate vectorial capacity [11,12,13,14] in disease vectors such as *Anopheles* and *Aedes*. Many factors, such as developmental stage, larval environment, arthropod host species, sex, pathogen infection and exposure to external chemicals, such as pesticides and antibiotics, can alter the composition of gut microbial communities of mosquito disease vectors [15,16,17,18,19]. 

The blood meal source was shown to have a significant impact on the microbial composition and diversity in the arthropod gut, but this remains largely unexplored. *Serratia*, a mosquito gut bacterium, was found to affect *Ae. aegypti* feeding behavior [20]. Through modulation of the chemosensory system, the microbiota also has the potential to influence the host-seeking behavior of arthropod disease vectors. The maternally transmitted microbiota influences odor emission and larval pheromone preference in *D. melanogaster* [21], thereby influencing food choice [22]. The modulatory effect of gut microbiota on the expression level of *vitellogenin* genes in the true bug *Riptortus pedestris* [23] and the involvement of *vitellogenin* expression in regulating host-seeking behavior of *Ae. albopictus* [24] indicates the possible role of the gut microbiome in the host-seeking behavior of mosquitoes. 

Recently, a strong influence of *Ae. aegypti* gut microbiota on the selection of the host blood meal was demonstrated [25]. Muturi et al., 2021 [25] observed a significant difference in alpha-diversity (Shannon index) of gut microbiota of laboratory-reared *Ae. aegypti* fed on either sugar, rabbit blood, or a mixture of chicken and rabbit blood, to that of chicken blood-fed mosquitoes. However, the impact of gut microbiota on the host preference in wild *Ae. aegypti* mosquitos collected from the field has yet to be investigated. The majority of previous studies used laboratory-reared mosquitos in controlled environments and did not employ human blood as a source of blood meal for the mosquitos. In this study, we used a 16S metagenomics approach to explore the gut microbiota of human-blood-fed and non-human-blood-fed versus non-fed wild-caught *Ae. Aegypti* and analyzed the association of any signature of gut microbial communities to the host blood meal preference.

## 2. Materials and Methods

### 2.1. Sample Collection and Processing of Samples

To control the bias due to incomplete penetrance [26], suitable sites were searched for *Ae. aegypti* mosquito collection within Bhopal city, Madhya Pradesh, India, where both cattle sheds and human houses co-exist for mosquito blood meal sources. Adult *Ae. aegypti* mosquitoes were collected by aspirator-based method from inside human houses as well as from cattle sheds. Mosquitoes were collected within the same season to control environment heterogeneity and its influence on host choice. Collected mosquitoes were stored immediately at 4 °C and transported to the laboratory. All collected mosquitoes were identified morphologically using a stereomicroscope following the taxonomic key described by Tyagi et al., 2015 [27].

**Figure 1:**
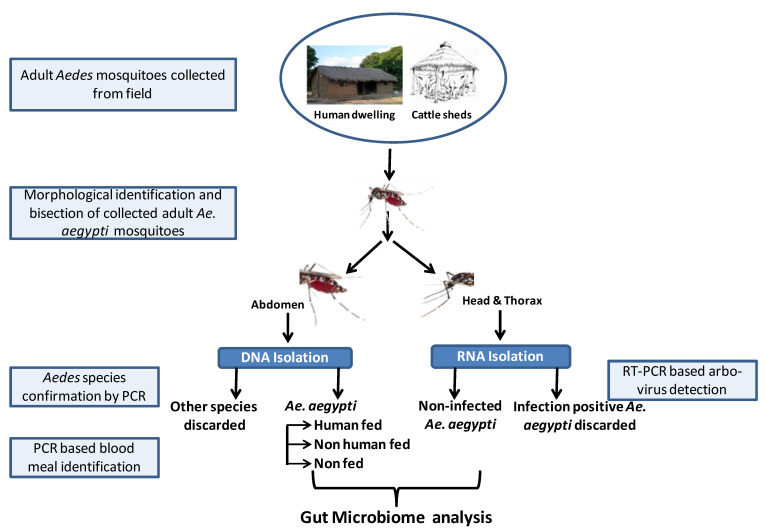
Description of the study design and experimental workflow.

Head, thorax and abdomen parts of all *Ae. Aegypti* mosquitoes were separated under a stereomicroscope using sterile forceps and needles. The individual head-plus-thorax and abdomen parts were put separately in sterile Dnase/RNAse-free 1.5 mL micro-centrifuge tubes and washed 2 times thoroughly with 100 µL 70% ethanol and then washed again with nuclease-free water. The separated parts of individual mosquitoes were stored in 50 uL of RNA later and preserved at −20 °C for further analysis, as illustrated in Figure 1. 

### 2.2. Nucleic Acid Extraction

DNA was extracted from abdomen part of individual *Ae. aegypti* mosquitoes using standard phenol–chloroform method. RNA was extracted from individual head–thorax part of each *Ae. aegypti* by using TriZol reagent. The quality of extracted DNA was assessed by running on agarose gel (0.75%) and nanodrop and quantity was determined using Qubit dsDNA HS kit (Invitrogen, Waltham, MA, USA, cat. No: Q32854) in Qubit4.0 fluorometer (Invitrogen). Similarly, the quality and quantity of the extracted RNA was assessed by using Qubit RNA IQ Assay kit (Invitrogen, Waltham, MA, USA, cat. No: Q33221) and Qubit RNA HS Assay Kit (Invitrogen, Waltham, MA, USA, cat. No: Q32852).

### 2.3. PCR Based Confirmation of Ae. aegypti, Identification of Host Blood Meal and Detection of Arbo-Viral Infection

Each morphologically identified *Ae. aegypti* mosquito was confirmed at molecular level using the DNA extracted from abdomen part following the method as described by Higa et al., 2010 [28]. 

We used a PCR-based approach to distinguish blood meal sources by specifically targeting the genetic material of host blood as the main inclusion criteria of samples for this study. Other blood meal identification approaches, such as the precipitin test or the enzyme-linked immunosorbent assays (ELISA) test, have limitations in identifying the host species due to low species specificity, use of live mosquitoes, and loss of biological material for further molecular studies. The PCR-based approach, on the other hand, has the advantage of being more specific, requiring less biological material, and being able to be used with degraded mosquitoes [29]. Source of host-blood meal was identified by using PCR-based method described by Field et al., 2020 [30]. This method differentiates human-blood-fed mosquitoes from non-human-blood-fed mosquitoes utilizing multiplex primer set targeting mitochondrial *cytochrome b* gene sequences. 

Given that gut microbiota can also modulate the pathogen infection status in mosquitoes [11,31], we screened the collected *Ae. aegypti* mosquitoes for prevalent arbovirus (DENV, CHIKV and ZIKV) infection using real-time PCR-based method [32]. RNA extracted from individual head–thorax was used for this purpose. 

PCR confirmed *Ae. aegypti* with known blood-fed status such as human-blood-fed (HF), non-human-blood-fed (NHF), and non-fed (NF) and devoid of any arbovirus infection with high-quality DNA (A_260_/A_280_ ratio >1.8) was used for further 16S metagenomics. 

### 2.4. Library Preparation and Sequencing

Samples were analyzed for bacterial taxonomy using the Ion 16S™ Metagenomics Kit according to the manufacturer’s instructions (Thermo Scientific, Waltham, MA, USA, cat no: A26216). Briefly, the 16S rDNA metagenomic library consisted of pooled and barcode adapters-tagged amplicons targeting V2–4–8 and V3–6, 7–9 sets; these two primer pools together enable for sequence-based identification of a wide range of bacteria in a mixed population [33]. Briefly, 10 ng of high-quality DNA was amplified using 2X Environmental Master Mix with the provided 10X 16S Primer Sets. PCR products were purified with AMPure^®^ XP beads solution (Beckman Coulter, Brea, CA, USA, cat no: A63881), quantified, pooled, and end-repaired using Ion Plus Fragment Library Kit (Thermo Scientific, cat no: 4471252). Then, amplicons were adapter-ligated, nick repaired, and quantified using an Ion Universal Library Quantitation Kit (Thermo Scientific, cat no: A26217). The library was pooled and diluted to the required concentration. The library template was prepared using Ion 520™ and Ion 530™ Kit–OT2 (Thermo Scientific, cat no: A27751) and was loaded on an Ion 530™ Chip (Thermo Scientific, cat no: A27763). Sequencing was performed at the central instrumentation facility of ICMR-National Institute for Research in Environmental Health on a GeneStudio S5 platform using Ion Torrent™ sequencing technology (Thermo Scientific, Inc., Waltham, MA, USA). Sequencing progress and raw sequences were processed through the Ion Torrent Suite (ver. 5.12.2, Thermo Fisher Scientific, Inc., Waltham, MA, USA), then BAM files were uploaded to the Ion Reporter Platform to obtain Ion 16S Metagenomics report through Ion 16S Metagenomics Workflow. Curation of raw sequence data and taxonomic assignment was carried out as described by Saeb et al., 2019 [34]. The 16S rRNA Operational Taxonomic Units (OTUs) were defined at ≥97% sequence homology. QIIME v2 [35] and the Greengenes reference dataset (ver. 3.18) from the Ribosomal Database Project (RDP) was used for all reads to classify to the lowest possible taxonomic rank. The criteria for bacterial species identification were: <97% identity for family and order level cut-off, >97% identity for genus level cut-off, and >99% identity for species-level cut-off.

### 2.5. Statistical Analyses

R version 3.3.2 [36] (https://cran.r-project.org/bin/windows/base/old/3.2.3/ accessed on 3 December 2021) was used for statistical analyses. The alpha-diversity metrics, including the Shannon and Simpson diversity index and observed OTUs (richness) were computed using the “*vegan*” package in R [37]. The “*vegan*” package was used to construct rarefaction curves to determine the sequencing coverage for each group of mosquitoes. Within and between-group differences in relative abundance of bacterial taxa were tested using Kruskal–Wallis (with post hoc Dunn test) and Wilcoxon rank-sum tests. A *p*-value < 0.05 was considered statistically significant. α-diversity indices and bacterial abundance between different groups were compared using one-way analysis of variance (ANOVA) followed by Dunn’s post hoc analysis and Bonferroni *p*-value corrections. Non-metric multidimensional scaling (NMDS) with Bray–Curtis similarity matrix values was conducted using the “vegan” package to assess the β-diversity and the NMDS plot was used to characterize within-group and between-group differences in bacterial communities. Stress values were used to assess the quality of NMDS representation; stress values <0.2 are considered a good representation of the data and those >0.3 are not considered to be valid [25]. The permutational multivariate analysis of variance (PERMANOVA) test with 9999 permutations was conducted in PAST v.3.14 [38] to determine the intra-group statistical differences of β-diversity. Differences in the microbial communities that were strongly associated with each blood-feeding behavior, was assessed using LEfSE (linear discriminatory analysis [LDA] effect size) to identify the unique bacterial taxa that drive differences between each of the groups [39] in the MicrobiomeAnalyst server [40]. Linear discriminant analysis (LDA) score threshold 2.0 and a *p*-value cut off at 0.05 were used. To remove technical bias related to different sequencing depths in different libraries, total sum scaling (TSS) normalization method was used in the MicrobiomeAnalyst server [40].

## 3. Results

**Figure 2:**
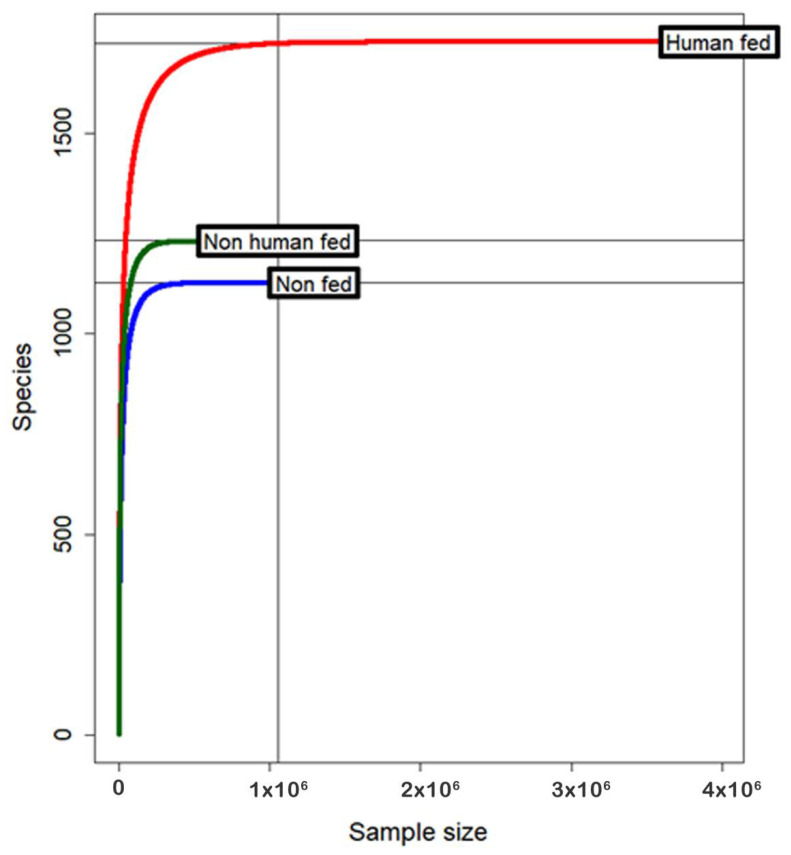
Rarefaction curve of the observed species.

A total of 279 adult female *Ae. aegypti* mosquitoes were collected in the peak season (between July and October 2019) from a site in Bhopal city where both cattle and humans reside on the same premise. After PCR-based confirmation of blood meal status, ruling out arbovirus infection using real-time PCR and based on the quality and quantity of extracted DNA, a total of 35 samples were processed for 16S metagenomics. These 35 mosquitoes contained three groups such as human-blood-fed (HF: *n* = 14), non-human-blood-fed (NHF: *n* = 11), and non-fed (NF: *n* = 10), respectively. A total of 23,198,192 reads were obtained after sequencing. After quality control, a total of 6,324,682 reads were mapped up to species level at >97% identity in the GreenGenes database. The mean number of reads per individual sample was 180,705.2  ±  SE 28,837.21 (Table 1). Sample wise mapped reads and numbers of attributed bacterial OUT are shown in Table S1. Rarefaction curves for the entire dataset were observed to reach a plateau, suggesting the microbial diversity present in *Ae. aegypti* mosquitoes was adequately recovered (Figure 2). 

A total of 2228 distinct bacterial OTUs were detected with a threshold of ≥97% similarity, belonging to 15 Phyla, 27 classes, 63 orders, 184 families, and 656 genera. The highest number of OTUs was observed in the HF *Ae. aegypti* mosquitoes (1729), followed by the NHF (1232) and the NF (1128) mosquitoes. Similarly, more genera, families and phyla were also observed for the HF mosquitoes compared to the NHF and the NF mosquitoes (Table 1). 

**Figure 3:**
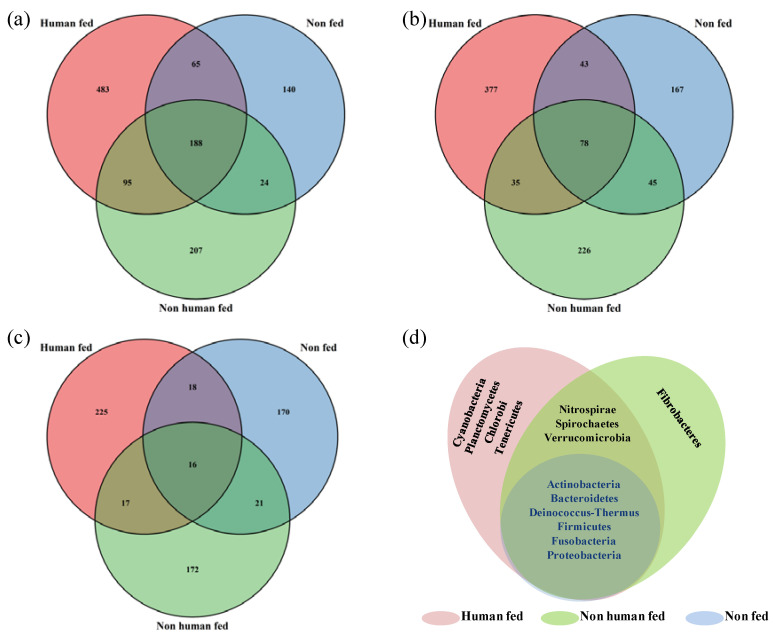
Venn diagrams showing the overlap of bacterial operational taxonomic units among human-fed, non-human-fed and non-fed mosquitoes (**a**) Species, (**b**) Genus, (**c**) Family and (**d**) Phylum.

Venn diagrams comparing the overlap of OTUs between the different mosquito groups revealed that 188 species, 78 genera, 16 families and 6 phyla were shared by the three groups. *Actinobacteria, Bacteroidetes, Deinococcus-Thermus, Firmicutes, Fusobacteria* and *Proteobacteria* were the six major phyla found exclusively at the core of all groups. Whereas, the number of unique OTUs associated with the HF *Ae. aegypti* mosquitoes were observed to be more at each level (Figure 3).

**Figure 4:**
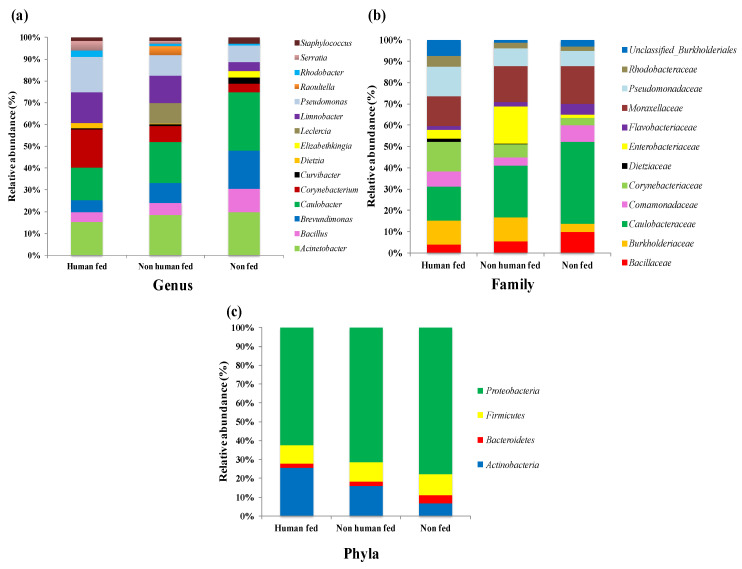
Relative abundance of most abundant bacterial taxa associated with human-fed, non-human-fed and non-fed mosquitoes (**a**) Genus, (**b**) Family and (**c**) Phyla.

Although 16 genera accounted for more than 70% of the total sequences, a differential abundance in the relative composition of these genera was observed. The abundance of *Corynebacterium* spp., *Pseudomonas* spp., *Serratia* spp. and *Dietzia* spp. was observed to be more in the HF (10.4%, 9.5%, 2.7% and 1.24%) mosquitoes in comparison to the NHF (4.75%, 6.1%, 0.9% and 0.1%) and the NF (2.76%, 5.5%, 0.01% and 0.05%) mosquitoes, respectively. On the contrary, the relative abundance of *Caulobacter* spp., *Acinetobacter* spp. and *Brevundimonas* spp. was higher in the NF (19.1%, 14.2% and 12.6%) in comparison to both the HF (9.0%, 8.8% and 3.3%) and the NHF (11.9%, 12.2% and 6.0%) mosquitoes, respectively. The genus *Leclercia* spp. was found exclusively in the NHF mosquitoes with relatively higher abundance (6.1%). Similarly, the abundance of the genus *Elizabethkingia* spp. was observed to be exclusively present in the NF mosquitoes (relative abundance: 2.03%) (Figure 4a). The differences in the relative abundance of major genus *Acinetobacter* spp., *Aquabacterium* spp., *Corynebacterium* spp., *Methylibium* spp., *Pseudomonus* spp. and *Rhodobacter* spp. was found to be significant between the HF and NHF mosquitoes. Similarly a significant difference in the relative abundance of bacteria of the genus *Aerococcus* spp., *Brevundimonas* spp., *Caulobacter* spp., *Dietzia* spp., *Micrococcus* spp. and *Rhodobacter* spp. was observed between the HF and NF mosquitoes (Table S2). Twelve major families were observed to account for more than 75% of the total sequences. Families such as *Burkholderiaceae* (8.5%), *unclassified*_*Burkholderiales* (5.6%), *Corynebacteriaceae* (10.4%) and *Dietziaceae* (1.2%) were highly abundant in the HF than the NHF (8.4%, 0.8%, 2.7% and 0.1%) and the NF (3.0%, 2.3%, 2.8% and 0.05%) mosquitoes, respectively. In the NF mosquitoes, the relative abundance of *Caulobacteraceae* (31.8%), *Moraxellaceae* (14.7%), *Bacillaceae* (8.2%) and *Flavobacteriaceae* (4.0%) were more abundant to that of the HF (12.2%, 10.4%, 2.9% and 1.3%) and the NHF (18.3%, 12.5%, 1.6% and 1.5%) mosquitoes, respectively. The family *Enterobacteriaceae* was found to be exclusively present in the NHF mosquitoes with 13.2% abundance than both the HF and NF mosquitoes (Figure 4b). At the phylum level, the dominance of both *Proteobacteria* and *Actinobacteria* was observed in all groups. However, the relative abundance of *Actinobacteria* was significantly more in the HF (25.5%) mosquitoes in comparison to that of the NHF (16.1%) (*p* < 0.05) and the NF (6.8%) mosquitoes. On the contrary, the relative abundance of *Proteobacteria* was found to be highest in the NF mosquitoes than the HF (*p* < 0.01) and NHF mosquitoes (77.8%, 71.1% and 62.3%, respectively) (Figure 4c). No significant difference was observed for *Proteobacteria* abundance between the NHF and NF mosquitoes (Table S2). 

Most of these differences were found to be significant when the relative abundance of the bacterial taxa of the HF mosquitoes was compared with that of the NHF and NF mosquitoes (Table S2), indicating a significant difference in the gut microbiota composition and abundancy of human-blood-fed *Ae. aegypti* mosquitoes. 

**Figure 5:**
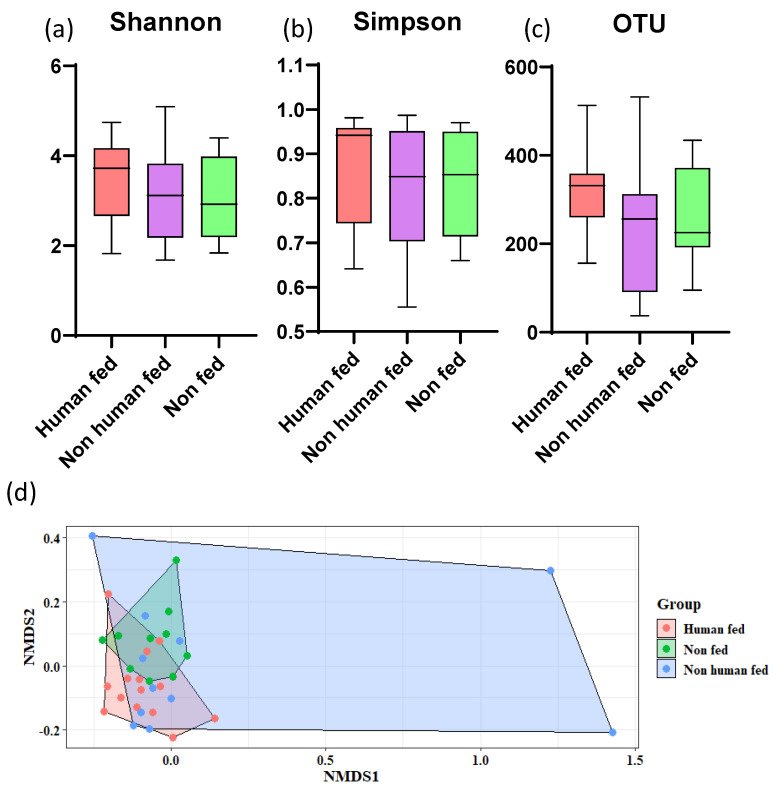
Distinct gut microbiome arrays in *Ae. aegypti* mosquitoes fed on human-and non-human-blood. (**a**–**c**) α-diversity indices; (**d**) β-diversity (NMDS analysis).

The overall low abundance of bacterial OTUs in NF mosquitoes was further observed in the α-diversity indices. Although the OTU richness, Shannon diversity, and Simpson diversity were observed to be higher in the HF mosquitoes than that of the NHF and NF *Ae. aegypti* mosquitoes (Figure 5a–c); however, these differences were non-significant. To further investigate if the three different groups corresponded to visibly differentiated structure of the gut microbiota of *Ae*. *aegypti*, we computed the *β*-diversity of the samples using NMDS with a Bray–Curtis similarity matrix. Although the NMDS did not cluster different groups into a distinct cluster, a stress value of 0.132 indicated the insignificant but noticeable effect of the blood-feeding behavior on the gut microbial communities of *Ae. aegypti* (Figure 5d). The PERMANOVA analyses, however, indicated that there was a significant difference in the gut microbiota content of the HF and NF mosquitoes (F = 5.8574, *p* = 0.03). 

To further identify differentially associated microbial communities within each group, we performed the microbiome biomarker discovery algorithm of LEfSe (Linear discriminatory analysis effect size) analysis to distinguish unique bacterial taxa in each group. The LDA (linear discriminatory analysis) score (Table 2) revealed significant differences in microbiota composition among the three groups. At the genus level, LEfSe indicated as many as 21 different bacterial genera differentially associated with the three groups (HF: 10, NHF: 5, NF: 6). *Ae. aegypti* mosquitoes that fed on human blood harbored a significantly higher proportion of *Rhodobacter*, *Rhizobacter*, *Rhodococcus*, *Gemmobacter*, *Ketogulonicigenium*, *Sphaerotilus*, *Malikia*, *Sphingobium*, *Dechloromonas* and *Yimella* compared to the other groups. On the other hand, mosquitoes that fed on non-human-blood harbored a high proportion of *Aerococcus*, *Frankia*, *Jeotgalibaca*, *Flaviflexus* and *Rhodoplanes*, whereas the genera *Caulobacter*, *Brevundimonas*, *Anoxybacillus*, *Millisia*, *Rhodoblastus* and *Parabacteroides* were found to be differentially associated with non-fed *Ae. aegypti* mosquitoes at a significant level. On the other hand, LEfSe identified six families differentially associated with the three groups. Of these, three families *Rhodobacteraceae, Neisseriaceae* and *Dermacoccaceae* were observed in higher abundance in the HF group. In contrast, two families (*Caulobacteraceae* and *Bradyrhizobiaceae*) and only one family (*Aerococcaceae*) were found to be significantly associated with the NF and NHF *Ae. aegypti* mosquitoes, respectively. At the class level, two classes (*Rhodobacterales* and *Neisseriales*) were found to be in higher abundance in the HF mosquitoes compared to a single class of bacteria (*Caulobacterales*) in the NF mosquitoes, with a relatively higher LDA score (Table 2).

## 4. Discussion

Dengue is a serious viral infection transmitted by dengue-virus-infected *Ae. aegypti* and *Ae. albopictus* mosquitoes. With an estimated global annual mortality of >104 million and morbidity of 40,000, more than half of humanity is under serious threat from dengue disease. Different control measures such as insecticide spray, environmental management, and source reduction of *Aedes* larvae are being widely used to control dengue infection. However, these control measures were somehow found less effective due to environmental heterogeneity, different bio-behavior of vector species and socio-economic conditions. Recently, alternative vector control strategies are in mainstay, among which the utilization of bacteria associated with different biological characteristics to be used as a potential control agent is one of the most promising strategies. In this approach, the mosquito endosymbiont bacteria, *Wolbachia*, has received significant attention due to its capacity to cause cytoplasmic incompatibility in female *Aedes* mosquitoes [41,42,43]. Similarly, the potential of other bacteria, such as *Serratia*, *Xenorhabdus* and *Photorhabdus*, colonizing the mosquito gut has been explored for disease vector control [44,45]. Therefore, there is an urgent need to examine the gut microbiota associated with disease vectors for use as a potential bio-control agent. 

The blood-feeding behavior and host preference is an epidemiologically important character for mosquitoes. The blood meal is required for the maturation of eggs but at the same time, the mosquito spreads pathogens to humans through which the disease is transmitted and maintained. As a result, host selection and blood-feeding behavior are critical, as a higher biting rate leads to an increased vectorial capacity as the vector has more opportunity to infect and transmit pathogens [4]. Recently, it was observed that the source of the mosquito blood meal has a strong influence on vector microbiota [25,46,47]. However, most of these studies were carried out in controlled laboratory conditions. To this end, we aimed to determine how human- and non-human blood meals affect the dynamics of the gut microbiota in *Ae. aegypti* mosquitoes collected from the actual environmental fields. We specifically designed the experiment in a way that both external factors could be controlled while maintaining the field condition. 

In the present study, we observed that the field-collected *Ae. aegypti* gut microbiota contained 2228 distinct bacterial species, which included 656 genera, 184 families, 63 orders, 27 classes, and 15 phyla. A higher gut microbiota composition of field-collected *Aedes* mosquitoes was also observed by others [48] in comparison to laboratory colonized mosquitoes [25,49,50]. These variations could be due to differences in the host blood meal source, habitat environment, geography and the methods used for gut microbiota detection (culture-based, 16S rRNA sequence-based or high throughput sequencing-based) [51]. The major phyla such as *Proteobacteria*, *Actinobacteria*, *Bacteroides*, and *Firmicutes* accounted for more than 95% of the total microbial community in the present study. These bacterial phyla were reported to occur most often by many of the studies in both laboratory-colonized *Ae. aegypti* [49,52]. However, within India, a higher prevalence of *Proteobacteria* (50%) and *Firmicutes* (30%) was observed in blood-fed *Ae. albopictus*, another established dengue vector, collected from Assam, India, using the culture method [53]. The higher abundance of members of *Enterobacteriaceae*, *Caulobacteraceae*, *Bacillaceae*, *Burkholderiaceae*, *Comamonadaceae*, *Flavobacteriaceae*, *Moraxellaceae* and *Pseudomonadaceae*, as observed in the present study, was also observed frequently in other studies with a variation in relative frequencies [49,52,53].

We observed a significantly higher abundance of bacterial OTUs associated with the HF mosquitoes in comparison to the NHF and NF mosquitoes (Table S2). The increased abundance of gut microbiota in *Aedes* mosquitoes may be due to the heme-mediated reduction of ROS activity inside the gut, which in terms proliferates the gut microbiota [54]. Some bacterial OTUs were observed to be exclusively associated with specific blood meal sources, indicating their potential role in the involvement of host choice behavior in *Ae. aegypti.* Although there are some OTUs that were commonly observed in all three groups, there is a significant difference in their relative abundance in each group. The exclusive abundance of the genus *Elizabethkingia* spp. (*Flavobacteriaceae*) in the NF mosquitoes support the findings from other studies where *Elizabethkingia* spp. was found in sugar-fed *Ae. Aegypti* females only [55] and with a lower abundance in *An. gambiae* after a blood meal [19]. 

Interestingly, the abundance of *Serratia* was observed to be very low in the HF group and almost negligible in the other two groups. As *Serratia* enhances the susceptibility of *Aedes* mosquitoes for dengue virus infection [11], and we used non-infected *Ae. aegypti* mosquitoes, our findings agree with those of other studies [20]. In the present study, no *Wolbachia* was identified in the 16S metagenomics sequences. Given that the prevalence of *Wolbachia* depends on the geography and environment, and that we collected all the mosquito samples from the same environment, along with the backdrop of no prior knowledge on the prevalence of *Wolbachia* in the study site, it would be difficult to confirm the presence of this endosymbiont species in Bhopal. 

Although the α-diversity and β-diversity among the three studies groups were found to be non-significant, a significant difference between the gut microbiota composition of the HF and NF *Ae. aegypti* mosquitoes was observed by the NMDS analysis. The differentially associated taxa based on LDA analysis also revealed OTUs consisting of as many as 21 genera, 6 families and 2 orders, which can be considered as a potential biomarker for the different blood-feeding behaviors of *Ae. aegypti.* However, the association of these bacterial taxa needs further investigation using a larger number of samples from different geographical origins to confirm their use as a biomarker species for host preference. 

In the present study, an abundance of the *Enterobacteriaceae* family was observed to be more in the NHF group in comparison to the HF and NF groups. However, contrary to our findings, Muturi et al., 2019 [25] observed a relatively higher abundance of this family in human-blood-fed mosquitoes. Members of the *Enterobacteriaceae* family, such as *Enterobacter* spp., are known to have a strong hemolytic activity, which helps in blood digestion and their abundance is known to increase after a blood meal due to their ability to strive in oxidative stress [19]. A significant association of members of the order *Rhodobacterales* such as *Rhodobacteraceae* and *Rhodobacter* spp. was observed with the HF mosquitoes (Table 2, Table S2). The abundance of *Rhodobacteraceae* increases the susceptibility of *Ae. aegypti* for Zika virus infection [52]. Recently Zika virus was incriminated in *Ae. aegypti* collected from Rajasthan [56], a neighboring state of Madhya Pradesh, implicating the importance of understanding the vector microbiota interaction in Zika virus disease epidemiology. Similarly a higher abundance of the members of the family *Dietziaceae*, specifically *Dietzia maris* and *Dietzia lutea*, was observed in the HF mosquitoes compared to that of NHF and NF mosquitoes. Recently *Dietzia maris* was isolated from *Ae. albopictus* and has great potential to be used as an insect paratransgenesis for the control of vector-borne diseases [57].

From the present study, it was also observed that the gut microbiota of the HF *Ae. aegypti* mosquitoes contains a large number of abundant human skin-associated bacteria [58] (Table 3), indicating a role of these bacteria in mosquito host selection. The differences in the skin microbiota composition of humans, other primates, and cattle could significantly modulate the mosquito host preference [59,60]. *Anopheles* mosquitoes were found to be attracted more towards people with a higher skin abundance of *Staphylococcus* spp. [59,61]. The human skin microbiota is thought to be a significant producer of volatile organic compounds (VOCs) and as many as 350 different VOCs are associated with human skin, of which many were demonstrated as a prominent mosquito attractant [62]. These compounds exhibit a clear difference in the VOC profile between and within animals including humans, suggesting the involvement of VOCs in anthropophilly (45). 

Being a nervous feeder, *Ae. aegypti* mosquitoes try to probe multiple times before a successful blood meal [63], which could result in microorganisms from diverse hosts being transported to the mosquito midgut. One of the most abundant human skin bacteria, *Corynebacteria,* was found to produce VOCs that attract malaria vector mosquitoes [64]. The observed higher abundance of *Corynebacteria* and *Staphylococcus* in the HF mosquitoes than that of the NHF and NF mosquitoes in the present study also indicates the microbiological basis of host preference in *Aedes* mosquitoes. Apart from these two bacteria, the abundance of other human skin-associated bacteria was also higher in the HF group, which may have prompted mosquitoes towards human blood. However, further, more comprehensive and inclusive studies are needed to validate this. 

The time after a blood meal also greatly influences the gut microbiota diversity of hematophagous mosquitoes. A substantial reduction (98%) of the midgut bacterial load was observed in *An. coluzzi* following a full blood meal and excretion of the blood bolus [65], which had been restored to pre-blood feeding levels by 72 h after a blood meal [18]. The restoration of gut microbiota after digestion of a blood meal was also observed by Muturi et al., 2019 [25] on *Ae. aegypti* mosquitoes that fed on human blood post- 3 and 7 days. However, the above studies were carried out either in the laboratory or in semi-field conditions where the timing of blood-feeding can be recorded precisely. As most of the arboviruses first establish infection in the midgut before spreading to various mosquito organs for lifelong infection, the virus still can modulate the gut microbial composition of the mosquito [31,66]. Therefore assaying viral infection in the midgut of the mosquito is also important to rule out the possible influence of pathogen infection on the observed gut microbial diversity.

The present study has some limitations. The extent of blood meal digestion in collected mosquitoes could not be assessed. The PCR-based method used in this study to identify the host blood meal source was specific with a lower detection limit of ~10 ng of host DNA [30] being expected to deliver the exact status of the blood meal source. In addition, the exact source of the non-human-blood meal was not carried out in this study, as we focused mainly on the differences in gut microbiota composition of human-blood-fed and non-human-blood-fed mosquitoes. Although we assessed the infection status of epidemiologically important viruses in the head and thorax part of the mosquitoes, the same was not performed in the midgut of the samples assessed for 16S metagenomics sequencing. For a better understanding of the role of gut microbiota on the host preference, these factors should be considered in further studies. 

## 5. Conclusions

To our knowledge, this is the first study to explore the gut microbiota composition of *Ae. aegypti* mosquitoes collected from field conditions with different blood meal sources. The study has several strengths, such as the amount of high-throughput sequencing coverage, identification of sufficient bacterial taxa (as revealed from the rarefaction curve), and the inclusion of non-infected mosquitoes. Overall, the findings reveal that the midgut microbiota of the human-blood-fed mosquitoes contains a significantly more diverse and higher abundance of bacterial flora in comparison to that of the non-human-blood-fed and non-fed mosquitoes. The gut microbial community of *Ae. aegypti* may be altered by the human-blood feeding. Further studies are needed to determine how changes in the midgut bacterial populations affect the vectorial capacity of *Aedes* mosquitoes in response to human-blood feeding by investigating the role of differentially associated bacterial taxa. 

## Figures and Tables

**Table 1:** Basic characteristics of the groups of *Ae. aegypti* mosquitoes based on 16S metagenomic sequences (N = numbers).

Groups	N, Mapped Reads	Reads per Sample, Mean ± SE	Range	N, Species	N, Genus	N, Family	N, Order	N, Class	N, Phylum	OTU Richness, Mean ± SE	Shannon Diversity, Mean ± SE	Simpson Diversity, Mean ± SE
Human-blood-fed	3,979,749	898,396 ± 515,266	1,533,754–217,166	1729	533	160	57	25	13	358.71 ± 29.25	5.75 ± 0.35	0.93 ± 0.03
Non-human-blood-fed	1,052,542	389,026 ± 179,033	723,077–183,328	1232	437	140	49	20	10	257.45 ± 48.75	4.17 ± 0.77	0.76 ± 0.06
Non-fed	1,292,391	634,137 ± 511,771	1,904,689–98,024	1128	385	131	44	16	6	270.90 ± 35.42	4.23 ± 0.64	0.82 ± 0.05
Total	6,324,682	180,705.2 ± 28,837.21		2228	656	184	63	27	15			

**Table 2:** Differentially abundant bacterial taxa that were significantly more abundant at log_10_ transformation in a given experimental group.

Taxonomic Position	Name	LDA Score	*p*-Value	Group
Order	*Caulobacterales*	5.99	0.020612	Non-fed
	*Rhodobacterales*	5.04	0.016629	Human-fed
	*Neisseriales*	3.85	0.01522	Human-fed
Family	*Caulobacteraceae*	5.99	0.020612	Non-fed
	*Rhodobacteraceae*	5.04	0.016629	Human-fed
	*Aerococcaceae*	4.92	0.018066	Non-human-fed
	*Neisseriaceae*	3.85	0.01522	Human-fed
	*Dermacoccaceae*	3.7	0.020067	Human-fed
	*Bradyrhizobiaceae*	3.51	0.038964	Non-fed
Genus	*Caulobacter*	5.71	0.0258	Non-fed
	*Brevundimonas*	5.66	0.014079	Non-fed
	*Aerococcus*	4.79	0.029434	Non-human-fed
	*Rhodobacter*	4.77	0.04871	Human-fed
	*Rhizobacter*	4.7	0.015001	Human-fed
	*Rhodococcus*	4.37	0.049157	Human-fed
	*Gemmobacter*	4.34	0.017545	Human-fed
	*Anoxybacillus*	4.12	0.012062	Non-fed
	*Ketogulonicigenium*	4.09	0.026514	Human-fed
	*Sphaerotilus*	3.99	0.0088411	Human-fed
	*Millisia*	3.61	0.0089651	Non-fed
	*Malikia*	3.54	0.037808	Human-fed
	*Frankia*	3.4	0.0085317	Non-human-fed
	*Rhodoblastus*	3.35	0.025347	Non-fed
	*Sphingobium*	3.34	0.049196	Human-fed
	*Jeotgalibaca*	3.3	0.030422	Non-human-fed
	*Parabacteroides*	3.23	0.016041	Non-fed
	*Dechloromonas*	3.09	0.03199	Human-fed
	*Rhodoplanes*	2.75	0.031148	Non-human-fed
	*Yimella*	2.6	0.037581	Human-fed
	*Flaviflexus*	2.45	0.031084	Non-human-fed

**Table 3:** Relative abundance of the most abundant human skin taxa in the three groups of *Ae. aegypti* mosquitoes.

Most Abundant Human Skin Taxa	Human-Fed	Non-Human-Fed	Non-Fed
*Propionibacterium acnes*	0.1946	0.0482	0.0351
*Corynebacterium tuberculostearicum*	0.0921	0.0367	0.0584
*Streptococcus mitis*	0.0315	0.0121	0.0286
*Streptococcus oralis*	0.0048	0.0005	0.0020
*Streptococcus pseudopneumoniae*	0.0018	0.0000	0.0003
*Streptococcus sanguinis*	0.0046	0.0022	0.0064
*Micrococcus luteus*	0.0012	0.0063	0.0000
*Staphylococcus epidermidis*	0.1065	0.0000	0.0000
*Staphylococcus capitis*	0.0012	0.0000	0.0000
*Veillonella parvula*	0.0000	0.0012	0.0205
*Staphylococcus hominis*	0.4192	0.1739	0.1423
*Corynebacterium fastidiosum*	0.0056	0.0000	0.0004
*Enhydrobacter aerosaccus*	0.0166	0.0011	0.0011
*Corynebacterium simulans*	0.0174	0.0006	0.0032
*Corynebacterium aurimucosum*	0.0003	0.0007	0.0010
*Corynebacterium amycolatum*	0.3400	0.0490	0.0469
*Staphylococcus warneri*	0.0942	0.0096	0.0109
*Staphylococcus haemolyticus*	0.0004	0.0000	0.0018
*Corynebacterium resistens*	1.2294	0.0263	0.0351
Total	2.5614	0.3686	0.3940

## Data Availability

All the raw sequencing datasets were submitted to the NCBI Sequence Read Archive database under SRA accession number: SUB10822463 and bio-project number PRJNA790716.

## Supplementary Materials

The following supporting information can be downloaded at: https://www.mdpi.com/article/10.3390/microorganisms10020332/s1, Table S1: Numbers of mapped reads and observed bacterial taxa in the *Aedes aegypti* mosquitoes (N = numbers); Table S2: Relative abundance (%) of the most prominent bacterial taxa associated with human-fed (HF), non-human-fed (NHF) and non-fed (NF) *Aedes aegypti* mosquitoes.
