# Supplementary material for: Influence of Host Blood Meal Source on Gut Microbiota of Wild Caught Aedes aegypti, a Dominant Arboviral Disease Vector
Source: Microorganisms. 2022 Feb 1;10(2):332. doi: 10.3390/microorganisms10020332 (PMC8880539; doi:10.3390/microorganisms10020332)
Supplement: Supplementary file 1 [file microorganisms-10-00332-s001.zip › microorganisms-1542462-supplementary.pdf]

**Table S1.** Numbers of mapped reads and observed bacterial taxa in the *Aedes aegypti* mosquitoes (N = numbers).

| Sample ID | N, Mapped Reads | N, Species | N, Genus | N, Family | N, Order | N, Class | N, Phylum |
|---|---|---|---|---|---|---|---|
| S_51 | 381558 | 363 | 160 | 80 | 32 | 15 | 8 |
| S_193 | 71472 | 354 | 162 | 78 | 33 | 13 | 5 |
| S_160 | 233650 | 233 | 128 | 128 | 31 | 14 | 6 |
| S_159 | 368118 | 401 | 223 | 92 | 38 | 15 | 6 |
| S_121 | 183005 | 237 | 148 | 71 | 30 | 13 | 7 |
| S_169 | 48981 | 181 | 117 | 59 | 23 | 10 | 4 |
| S_126 | 740787 | 287 | 173 | 80 | 30 | 14 | 5 |
| S_186 | 249173 | 462 | 249 | 101 | 38 | 17 | 7 |
| S_178 | 118283 | 372 | 231 | 99 | 37 | 15 | 5 |
| S_175 | 140777 | 310 | 179 | 85 | 33 | 16 | 6 |
| S_87 | 412792 | 287 | 154 | 75 | 29 | 14 | 5 |
| S_107 | 24405 | 138 | 90 | 48 | 23 | 9 | 4 |
| S_22 | 321032 | 307 | 169 | 82 | 34 | 17 | 8 |
| S_28 | 685716 | 300 | 164 | 73 | 29 | 14 | 5 |
| S_92 | 52771 | 349 | 165 | 79 | 34 | 15 | 6 |
| S_106 | 123018 | 385 | 181 | 85 | 29 | 14 | 5 |
| S_104 | 15875 | 95 | 49 | 32 | 16 | 9 | 4 |
| S_148 | 104255 | 100 | 48 | 18 | 10 | 6 | 4 |
| S_02 | 232945 | 607 | 257 | 102 | 38 | 17 | 7 |
| S_161 | 8254 | 40 | 29 | 16 | 10 | 5 | 3 |
| S_132 | 130875 | 275 | 137 | 63 | 27 | 13 | 5 |
| S_59 | 85698 | 266 | 127 | 62 | 29 | 14 | 7 |
| S_88 | 123075 | 166 | 86 | 46 | 19 | 9 | 6 |
| S_122 | 75390 | 333 | 158 | 83 | 29 | 13 | 5 |
| S_42 | 100386 | 216 | 121 | 59 | 28 | 13 | 5 |
| S_55 | 120631 | 391 | 183 | 86 | 31 | 13 | 5 |
| S_30 | 151154 | 399 | 182 | 84 | 35 | 15 | 6 |
| S_19 | 114344 | 206 | 103 | 62 | 31 | 13 | 5 |
| S_40 | 27900 | 216 | 120 | 70 | 30 | 15 | 6 |
| S_58 | 144690 | 461 | 199 | 94 | 35 | 16 | 6 |
| S_167 | 145536 | 242 | 120 | 61 | 26 | 10 | 5 |
| S_163 | 44574 | 96 | 61 | 38 | 21 | 8 | 4 |
| S_25 | 62345 | 264 | 127 | 64 | 28 | 11 | 5 |
| S_155 | 321540 | 190 | 101 | 51 | 22 | 9 | 4 |
| S_36 | 159677 | 244 | 126 | 63 | 25 | 11 | 5 |

**Table S2.** Relative abundance (%) of the most prominent bacterial taxa associated with human-fed (HF), non human-fed (NHF) and non-fed (NF) *Aedes aegypti* mosquitoes.

| Taxa | HF | NHF | NF | *p*-Values of within Group Differences | *p*-Values of between Group Differences | | |
|---|---|---|---|---|---|---|---|
| | | | | | HFvsNHF | HFvsNF | NHFvsNF |
| | | | | **Phylum** | | | |
| *Actinobacteria* | 25.53 | 16.06 | 6.77 | **0.029** | **0.032** | 0.065 | 0.232 |
| *Bacteroidetes* | 2.45 | 2.30 | 4.42 | 0.074 | 0.083 | 0.846 | 0.625 |
| *Deinococcus-Thermus* | 0.08 | 0.26 | 0.05 | 0.077 | 0.275 | 0.477 | 0.570 |

| | | | | | | | |
|---|---|---|---|---|---|---|---|
| *Fibrobacteres* | 0.00 | 0.00 | 0.00 | 0.201 | 0.083 | 0.492 | 0.999 |
| *Proteobacteria* | 62.26 | 71.14 | 77.78 | **0.0001** | **0.001** | **0.002** | 0.193 |
| **Family** | | | | | | | |
| *Acetobacteraceae* | 1.01 | 0.60 | 0.31 | 0.170 | 0.065 | 0.084 | 0.477 |
| *Aerococcaceae* | 1.08 | 2.23 | 0.48 | **0.024** | 0.206 | **0.009** | 0.232 |
| *Bacillaceae* | 2.93 | 4.02 | 8.17 | 0.079 | 0.123 | 0.695 | 0.106 |
| *Bartonellaceae* | 0.24 | 1.09 | 0.00 | 0.439 | 0.438 | 0.125 | 0.500 |
| *Burkholderiaceae* | 8.46 | 8.53 | 3.04 | 0.170 | 0.148 | 0.232 | 0.193 |
| *Caulobacteraceae* | 12.17 | 18.28 | 31.77 | **0.019** | 0.831 | 0.084 | 0.193 |
| *Chromatiaceae* | 0.87 | 1.19 | 1.36 | 0.096 | 0.206 | 0.999 | 0.232 |
| *Clostridiales* | 0.53 | 0.15 | 0.06 | 0.072 | **0.014** | 0.064 | 0.734 |
| *Comamonadaceae* | 5.32 | 2.72 | 6.31 | **0.020** | **0.014** | 0.106 | 0.556 |
| *Corynebacteriaceae* | 10.36 | 4.75 | 2.76 | 0.101 | **0.042** | 0.131 | 0.625 |
| *Dietziaceae* | 1.24 | 0.15 | 0.05 | 0.169 | 0.322 | **0.019** | 0.312 |
| *Enterobacteriaceae* | 3.14 | 13.19 | 1.22 | 0.599 | 0.898 | 0.769 | 0.625 |
| *Flavobacteriaceae* | 1.29 | 1.59 | 4.05 | 0.164 | 0.102 | 0.695 | 0.769 |
| *Geodermatophilaceae* | 0.18 | 0.54 | 0.13 | 0.069 | **0.054** | 0.065 | 0.922 |
| *Hyphomicrobiaceae* | 0.61 | 0.04 | 0.21 | **0.001** | **0.009** | 0.064 | **0.039** |
| *Intrasporangiaceae* | 0.32 | 1.48 | 0.12 | **0.030** | 0.577 | **0.014** | 0.160 |
| *Methylophilaceae* | 0.50 | 0.70 | 0.65 | 0.461 | 0.175 | 0.652 | 0.999 |
| *Microbacteriaceae* | 0.56 | 0.86 | 0.12 | **0.005** | 0.206 | **0.027** | 0.496 |
| *Micrococcaceae* | 3.19 | 2.23 | 2.05 | **0.030** | **0.032** | 0.480 | 0.557 |
| *Moraxellaceae* | 10.39 | 12.50 | 14.67 | 0.220 | **0.042** | 0.492 | 0.490 |
| *Nocardioidaceae* | 0.67 | 0.47 | 0.38 | **0.033** | 0.080 | **0.040** | 0.999 |
| *Planococcaceae* | 0.34 | 0.59 | 0.10 | 0.169 | 0.278 | 0.064 | 0.160 |
| *Propionibacteriaceae* | 0.94 | 1.16 | 1.02 | 0.489 | 0.413 | 0.846 | 0.922 |
| *Pseudomonadaceae* | 10.53 | 6.35 | 5.87 | **0.040** | **0.042** | 0.375 | 0.922 |
| *Pseudonocardiaceae* | 0.57 | 1.42 | 0.29 | **0.042** | 0.067 | **0.006** | 0.232 |
| *Rhodobacteraceae* | 3.82 | 2.00 | 1.76 | **0.003** | **0.019** | **0.014** | 0.846 |
| *Sinobacteraceae* | 1.58 | 0.87 | 1.83 | 0.136 | 0.700 | 0.275 | 0.820 |
| *Sphingomonadaceae* | 0.58 | 0.84 | 0.97 | 0.114 | 0.240 | 0.432 | 0.625 |
| *Staphylococcaceae* | 1.57 | 1.57 | 2.20 | 0.791 | 0.175 | 0.556 | 0.322 |
| *Unclassified_Burkholderiales* | 5.55 | 0.79 | 2.33 | **0.015** | **0.032** | 0.769 | 0.432 |
| *Xanthomonadaceae* | 1.01 | 0.79 | 0.63 | 0.129 | **0.019** | 0.160 | 0.922 |
| **Genus** | | | | | | | |
| *Acinetobacter* | 9.02 | 11.95 | 14.17 | 0.238 | **0.032** | 0.625 | 0.492 |
| *Aerococcus* | 0.75 | 1.60 | 0.32 | **0.024** | 0.148 | **0.004** | 0.275 |
| *Aquabacterium* | 3.76 | 0.57 | 2.18 | **0.018** | **0.024** | 0.846 | 0.232 |
| *Bacillus* | 2.56 | 3.66 | 7.55 | 0.062 | 0.206 | 0.695 | 0.135 |
| *Bartonella* | 0.24 | 1.09 | 0.00 | 0.439 | 0.437 | 0.125 | 0.500 |
| *Brevundimonas* | 3.34 | 6.03 | 12.62 | **0.014** | 0.765 | **0.049** | 0.232 |
| *Caulobacter* | 8.77 | 12.20 | 19.07 | **0.023** | 0.966 | **0.025** | 0.131 |
| *Comamonas* | 0.59 | 0.14 | 0.09 | 0.093 | 0.084 | 0.084 | >0.999 |
| *Corynebacterium* | 10.36 | 4.75 | 2.76 | 0.100 | **0.042** | 0.131 | 0.625 |
| *Curvibacter* | 0.31 | 0.57 | 1.94 | 0.237 | 0.123 | 0.203 | >0.999 |
| *Dietzia* | 1.24 | 0.15 | 0.05 | 0.169 | 0.322 | **0.020** | 0.312 |
| *Elizabethkingia* | 0.00 | 0.00 | 2.03 | NA | NA | NA | NA |
| *Enhydrobacter* | 0.59 | 0.25 | 0.30 | **0.009** | **0.019** | **0.020** | 0.486 |
| *Enterobacter* | 0.04 | 1.75 | 0.04 | 0.173 | 0.846 | 0.064 | 0.172 |
| *Facklamia* | 0.28 | 0.61 | 0.13 | 0.225 | 0.638 | 0.084 | 0.074 |
| *Flavobacterium* | 0.76 | 0.95 | 1.71 | 0.171 | **0.024** | 0.922 | 0.232 |
| *Gemmobacter* | 0.57 | 0.14 | 0.16 | **0.020** | **0.024** | 0.084 | 0.695 |

| | | | | | | | |
|---|---|---|---|---|---|---|---|
| *Hydrogenophaga* | 1.20 | 0.05 | 0.10 | **0.032** | **0.027** | 0.787 | 0.262 |
| *Klebsiella* | 0.04 | 0.64 | 0.10 | 0.352 | 0.131 | >0.999 | 0.297 |
| *Kocuria* | 1.36 | 0.72 | 1.10 | **0.018** | **0.014** | **0.027** | 0.232 |
| *Leclercia* | 0.13 | 6.14 | 0.02 | 0.966 | 0.945 | 0.164 | 0.887 |
| *Limnobacter* | 8.28 | 8.24 | 2.97 | 0.115 | 0.102 | 0.275 | 0.232 |
| *Methylibium* | 2.11 | 1.06 | 2.57 | 0.189 | **0.042** | 0.557 | 0.695 |
| *Methylophilus* | 0.40 | 0.66 | 0.03 | 0.139 | 0.131 | 0.496 | >0.999 |
| *Methylotenera* | 0.09 | 0.05 | 0.62 | 0.679 | 0.578 | 0.469 | 0.516 |
| *Micrococcus* | 1.59 | 1.30 | 0.74 | **0.044** | 0.067 | **0.019** | 0.275 |
| *Nevskia* | 1.49 | 0.85 | 1.72 | **0.004** | 0.067 | 0.084 | 0.193 |
| *Nocardioides* | 0.59 | 0.44 | 0.36 | **0.044** | 0.067 | **0.037** | 0.769 |
| *Paracoccus* | 0.87 | 0.78 | 0.64 | 0.053 | **0.014** | 0.105 | 0.275 |
| *Piscinibacter* | 0.61 | 0.00 | 0.02 | 0.200 | **0.031** | 0.460 | 0.297 |
| *Propionibacterium* | 0.89 | 1.13 | 0.99 | 0.493 | 0.365 | >0.999 | 0.922 |
| *Pseudomonas* | 9.50 | 6.09 | 5.46 | 0.065 | **0.042** | 0.232 | 0.846 |
| *Pseudonocardia* | 0.37 | 1.02 | 0.24 | 0.207 | 0.067 | **0.009** | 0.375 |
| *Raoultella* | 0.01 | 2.63 | 0.00 | 0.509 | 0.688 | 0.625 | 0.312 |
| *Rheinheimera* | 0.87 | 1.19 | 1.36 | 0.096 | 0.206 | >0.999 | 0.232 |
| *Rhizobacter* | 0.99 | 0.02 | 0.01 | 0.051 | **0.019** | **0.039** | 0.844 |
| *Rhodobacter* | 1.72 | 0.67 | 0.60 | **0.008** | **0.019** | **0.027** | >0.999 |
| *Rubrivivax* | 0.89 | 0.20 | 0.13 | 0.224 | 0.147 | 0.084 | 0.734 |
| *Serinicoccus* | 0.14 | 1.00 | 0.05 | **0.023** | 0.413 | **0.027** | 0.148 |
| *Serratia* | 2.66 | 0.94 | 0.01 | 0.493 | >0.999 | 0.562 | >0.999 |
| *Sphingomonas* | 0.33 | 0.64 | 0.72 | 0.163 | 0.175 | 0.695 | 0.637 |
| *Staphylococcus* | 0.92 | 1.04 | 2.04 | 0.354 | 0.413 | 0.922 | 0.193 |
| *Zavarzinia* | 0.55 | 0.05 | 0.00 | 0.110 | 0.937 | 0.125 | 0.500 |

**Bold:** Indicates statistically significant $p$-values < 0.05; NA: Statistical tests not performed due to exclusive presence of the genus in only one group.
